# Neuroblastoma metastasized to the mandible and the spinal extradural regions: A case report

**DOI:** 10.1097/MD.0000000000024399

**Published:** 2021-04-30

**Authors:** Xiaomei Sun, Hui Xu, Kai Xin, Bo Sun

**Affiliations:** a Cancer Center; b Department of Ophtalmology, The First Hospital of Zibo, Zibo, Shandong, China.

**Keywords:** metastasis, Neuroblastoma, radiotherapy

## Abstract

Neuroblastoma (NB) metastasized into the mandible and spinal extradural region was rarely reported. We present a case with metastatic NB to the mandible and the spinal extradural regions. The patient received chemotherapy using NB 97 regimen and was tumor free after 8 months, but 9 months after the treatment, the patient presented with lower limb paralysis and persistent pain in maxillofacial region as well as swelling in the left mandibular area. Metastasis to the mandible and the spinal extradural regions was diagnosed based on the spinal and maxillofacial magnetic resonance imaging. Radiotherapy with a density of 2 Gy per day was given via a linear accelerator. The total dose of the intraspinal occupying lesion was subject to radiotherapy with a regimen of 30 Gy (10 fractions). For the management of the maxillofacial pain, tumor in the maxillofacial region was subject to a density of 50 Gy (25 fractions). The maxillofacial pain disappeared after a density of 10 Gy and soft tissue tumefaction was eliminated after a density of 50 Gy, and the maxillofacial appearance was much better than before. Finally, the patient died from tumor progression 2 years after diagnosis for NB.

## 1 Introduction

Neuroblastoma (NB) derived from neural crest cells of the adrenal medulla or sympathetic ganglia^[1]^ is responsible for 15% of cancer-related death in children. In clinical practice, the criterion standard for NB diagnosis is histology based on biopsy.^[2]^ The symptoms were usually related to the location of the primary or metastatic lesions, such as malaise, abdominal mass and pain, back pain, fever, anemia, and weight loss. Currently, the treatment of NB is depending on the staging by International Neuroblastoma Staging System (INSS) or International Neuroblastoma Risk Group (INRG), and usually involves a combination of surgery, chemotherapy, and/or radiation.^[2]^ Unfortunately, many patients showed poor outcome after the treatment due to metastasis. NB metastasis via both hematogenous and lymphatic routes may spread to a variety of locations including lymph nodes, bone marrow, cortical bone, dura, the orbits, liver, and skin.^[3]^ However, rare cases with NB metastasized into the mandible and spinal extradural region have been reported. In this case, we report a child suffering from metastatic NB to the mandible and the spinal extradural areas.

## 2 Case presentation

A 4-year-old female child presented to the Pediatric Department of our hospital due to anemia lasting for 1 month. Abdominal ultrasonography revealed a medium echo area measuring 68.8 × 44.5 × 76.8 mm. Abdominal computed tomography (CT) showed a mass (6.8 cm in size) with irregularly shape in the right adrenal gland. The thickness of the renal parenchyma was not even. Chest CT showed pleural effusion and thickening, as well as thoracic bone destruction.

**Figure 1:**
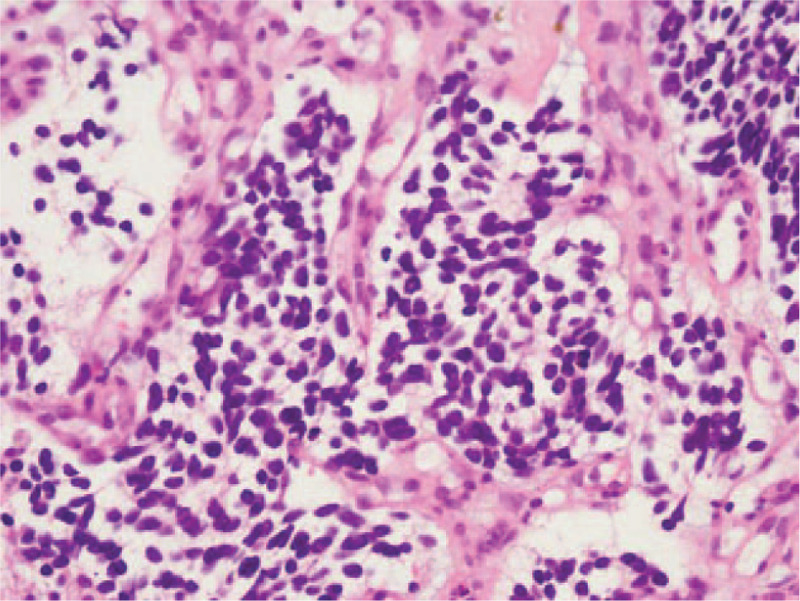
Histological slide (HE, 400×). Histological examination of the tumor revealed dense proliferation of small round tumor cells with sparse cytoplasm and hyperchromatic nuclei.

Right adrenal gland biopsy and bone marrow biopsy were carried out to establish a definitive histological diagnosis, which revealed poorly differentiated NB. Stage IV NB was diagnosed based on the histology, INSS, or INRG. Hematoxylin and eosin staining revealed dense proliferation of small round tumor cells with sparse cytoplasm and hyperchromatic nuclei (Fig. 1).

For the treatment, chemotherapy was given using German NB 97 regimen which was consisted of 6 cycles of chemotherapy with some dose reductions (cisplatinum 24 hours 40 mg/m^2^ per day days 1–4 [96 h], etoposide 24 hours 100 mg/m^2^ per day days 1 to 4, vindesine 1 hour 3 mg/m^2^ per day 1; doxorubicine 4 hours, 30 mg/m^2^ per day, days 6–7) to complete within 5 months according to the previous description.^[4]^ Meanwhile, abdominal surgery was performed to remove tumor remnants, followed by radiotherapy (DT: 40 Gy/25 F). Afterwards, the patient received 3-month maintenance chemotherapy (cyclophosphamide oral 150 mg/m^2^ per day for days 1–7; 4 cycles). Eventually, complete remission was obtained.

**Figure 2:**
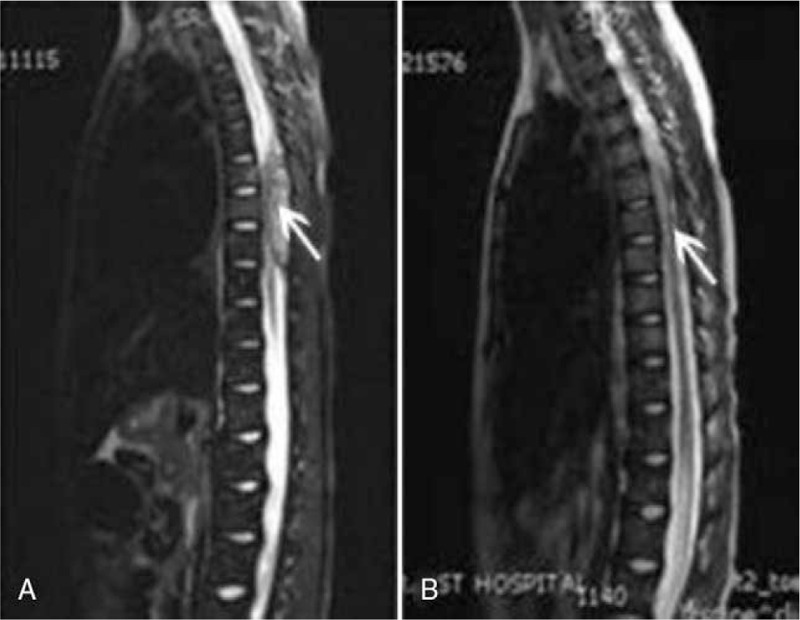
Spinal magnetic resonance imaging revealed a dorsal epidural mass from the level of T6 to T8 before and after radiotherapy.

**Figure 3:**
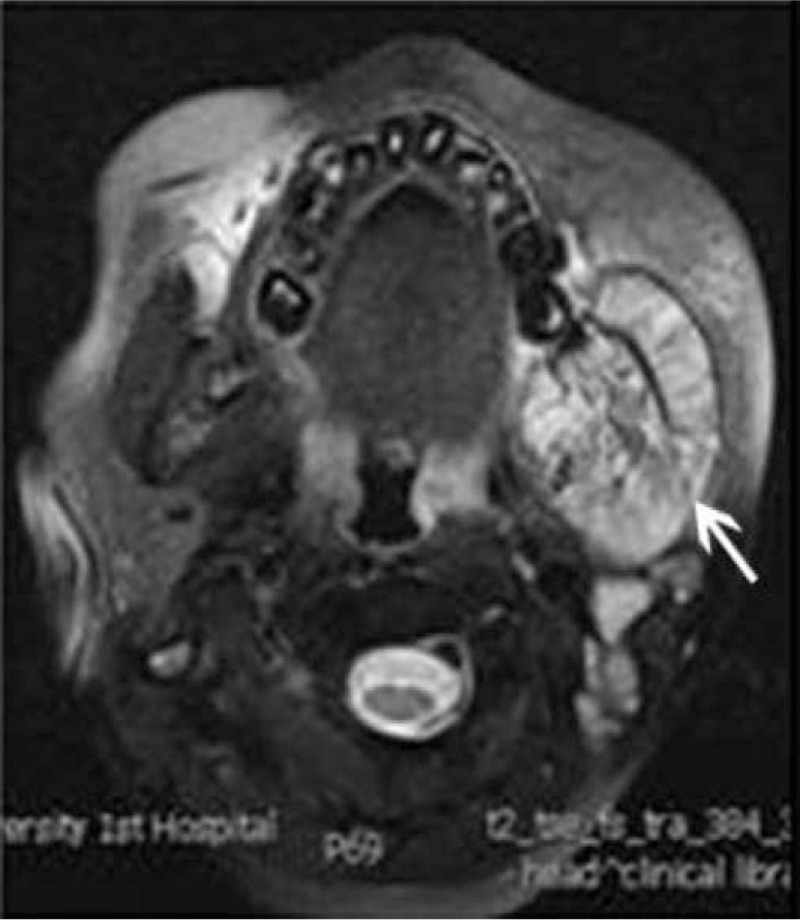
Magnetic resonance imaging scan showed extension of a mass in the left side of the mandible with a clear osseous destruction before radiotherapy.

The patient is tumor-free after 8-month-follow up. Nevertheless, 9 months after the treatment, the patient presented with lower limb paralysis and persistent pain in maxillofacial region as well as swelling in the left mandibular area. She presented to our department with progressive paraparesis, urinary retention, and hypoesthesia. Her muscle strength was fair to good in the upper extremities but nonexistent in all muscles of the lower extremities at admission. Lower limb reflexes were pathologically brisk. Spinal magnetic resonance imaging (MRI) revealed a dorsal epidural mass from T6 to T8 levels (Fig. 2A). Axial imaging was obtained using MRI. Maxillofacial MRI displayed longer T1 signals together with whole tumor inhomogeneous enhancement and destruction of the cortical bony (Fig. 3). On this basis, the patient was diagnosed with metastasis to the mandible and the spinal extradural regions.

**Figure 4:**
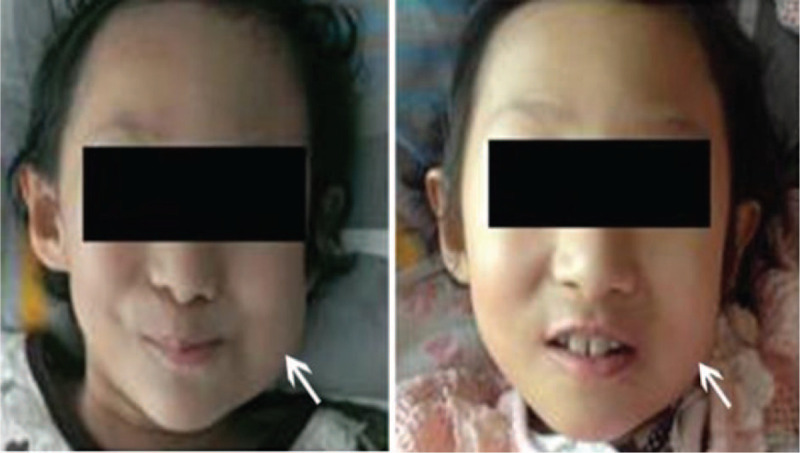
Maxillofacial appearance before (left) and after (right) radiotherapy.

After taking the previous treatment dose and the organs at risk into consideration, radiotherapy with a density of 2 Gy per day was given via a linear accelerator (6 MV photons). For the management of intraspinal tumor, the total dose of the intraspinal occupying lesion was subject to radiotherapy with a regimen of 30 Gy × 10 fractions. To relieve the maxillofacial pain, the tumor in the maxillofacial region was subject to a density of 50 Gy × 25 fractions. The maxillofacial pain disappeared after a density of 10 Gy and soft tissue tumefaction was eliminated after a density of 50 Gy, and maxillofacial appearance was much better than before (Fig. 4). MRI indicated a sharp decrease of tumor size after a density of 30 Gy in spinal extradural region (Fig. 2B). Finally, the patient died from tumor progression 2 years after diagnosis for NB.

## 3 Discussion

Dissemination of NB to bones, skull, and lymph nodes has been well described^[5,6]^; however, rare cases showed involvement in the head and neck region.^[7,8]^ In this case, we presented a rare case of an adrenal NB with metastasis to the mandible and spinal extradural regions.

The incidence of the disease was 10.5 per 1 million children aged <15 annually.^[9]^ Localization and histopathological characteristics may vary among NB patients.^[9]^ The disease is known for its wide variety of clinical manifestations. NB metastasis by both hematogenous and lymphatic routes and my spread to a wide variety of locations including lymph nodes, bone marrow, cortical bone, dura, the orbits, liver, and skin.^[3]^ Moreover, mandible and spinal metastasis is rare in general. In this study, a literature review was performed to summarize the cases of mandible metastasis, together with the treatment regimen and outcome (Table 1).^[3,8,10–16]^

**Table 1:** Summary of NB patients with metastasis to the mandible.

Patient no.	Age at diagnosis at NB/sex	Primary site	Metastases	Treatment	Outcomes	References no.
1	7 y/M	Adrenal	Left body of the mandible	C + S	Died 8 mo after diagnosis	^[10]^
2	5 y/M	Adrenal	Right side of the mandible	C + R	NA	^[11]^
3	2.5 y/M	Adrenal	Mandibular vertebral bodies	C + R	Died 5 mo after diagnosis	^[12]^
4	2.5 y/M	Left side of the mandible	left side of the mandible	C + R	NA	^[13]^
5	8 y/F	NA	Mandible	R	Died 24 mo after diagnosis	^[14]^
6	1 y/F	NA	Mandible	R	Died 13 mo after diagnosis	^[14]^
7	1.5 y/F	NA	Mandible	R	Died 29 mo after diagnosis	^[14]^
8	1.5 y/F	NA	Mandible	R	Died 5 mo after diagnosis	^[14]^
9	2 y/F	NA	Mandible	R	Died 8 mo after diagnosis	^[14]^
10	3 y/M	NA	Mandible	R	Died 18 mo after diagnosis	^[14]^
11	3 y/M	Left adrenal	The left mandible	C	Died 1 mo after diagnosis	^[8]^
12	15 y/F	NA	The left mandible	C	NA	^[3]^
13	2 y/M	NA	mandibular condyle	S + C + stem cell rescue	NA	^[15]^
14	8 mo/M	At the superior pole of the right kidney	The left mandible	S + C	2 y After well	^[16]^

C = chemotherapy, F = female, M = male, NA = not available, NB = neuroblastoma, R = radiotherapy, S = surgery.

Up to now, no approved standard guidelines have been established for treating NB. The treatment of NB is mainly depending on the surgery, radiation therapy, chemotherapy, bone marrow transplantation, and immunotherapy.^[17–19]^ In the literature review, 14 cases with NB metastasized to the mandible region were included, and the treatment was highly depending on the chemotherapy, surgery and radiotherapy. In this case, the patient showed metastatic NB to the mandible and the spinal extradural areas, and radiotherapy is a treatment option for the disease after taking the previous treatment dose and the organs at risk into consideration.

In this case, we reported a case of metastatic NB to the mandible and the spinal extradural areas. The radiotherapy may serve as a treatment option. In future, further studies are needed to validate the efficiency of radiotherapy in the NB metastasis to the mandible and the spinal extradural areas.

## Author contributions

**Conceptualization:** Hui Xu.

**Data curation:** Hui Xu.

**Formal analysis:** Hui Xu.

**Resources:** Kai Xin.

**Software:** Kai Xin.

**Supervision:** Kai Xin.

**Writing – original draft:** Xiaomei Sun.

**Writing – review & editing:** Bo Sun.

## Corrections

This article originally published incorrectly under the article type Observational Study and has since been corrected to Case Report.

## References

[1] Sul HJ Kang D. Congenital neuroblastoma with multiple metastases: a case report. J Korean Med Sci 2003;18:618–20. 12923347 10.3346/jkms.2003.18.4.618 PMC3055087

[2] Zhu J Hoag NA Gustafson P. Pediatric bladder neuroblastoma: case report and literature review. Can Urol Assoc J 2013;7:E609–611. 24069107 10.5489/cuaj.183 PMC3776040

[3] Baber MA Abubaker O Laskin DM. Metastatic neuroblastoma in the mandibular condyle: report of a case. J Oral Maxillofac Surg 2008;66:1941–5. 18718405 10.1016/j.joms.2007.06.689

[4] Berthold F Hero B Kremens B. Long-term results and risk profiles of patients in five consecutive trials (1979-1997) with stage 4 neuroblastoma over 1 year of age. Cancer Lett 2003;197:11–7. 12880954 10.1016/s0304-3835(03)00076-4

[5] Ogawa T Hara K Kawarai Y. A case of infantile neuroblastoma with intramucosal metastasis in a paranasal sinus. Int J Pediatr Otorhinolaryngol 2000;55:61–4. 10996238 10.1016/s0165-5876(00)00379-7

[6] Pellegrino SV TR B. Expansile mandibular lesion in a child. Oral Surg Oral Med Oral Pathol Oral Radiol Endodont 2000;90:135–9. 10.1067/moe.2000.107053 10936830

[7] Haddad M Triglia JM Helardot P. Localized cervical neuroblastoma: prevention of surgical complications. Int J Pediatr Otorhinolaryngol 2003;67:1361–7. 14643482 10.1016/j.ijporl.2003.08.046

[8] Otmani N Khattab M. Metastatic neuroblastoma to the mandible in a 3-year-old boy: a case report. Med Oral Patol Oral Cir Bucal 2007;12:E201–204. 17468714

[9] Csanady M Vass G Bartyik K. Multidisciplinary management of cervical neuroblastoma in infants. Int J Pediatr Otorhinolaryngol 2014;78:2103–6. 25306308 10.1016/j.ijporl.2014.09.015

[10] Manor E Kapelushnik J Joshua BZ. Metastatic neuroblastoma of the mandible: a cytogenetic and molecular genetic study. Eur Arch Otorhinolaryngol 2012;269:1967–71. 22134668 10.1007/s00405-011-1863-9

[11] Hardt NP. Metastatic neuroblastoma in the mandible. report of a case. Oral Surg Oral Med Oral Pathol 1976;41:314–20. 1061918 10.1016/0030-4220(76)90144-4

[12] Boraz RA. Neuroblastoma: case involving metastases to the mandible. Pediatr Dent 1985;7:315–7. 3868770

[13] Borle RM Hazare VK Bhowate RR. Neuroblastoma metastatic to the mandible. J Oral Maxillofac Surg 1991;49:1124–6. 1890526 10.1016/0278-2391(91)90150-k

[14] Deutsch M Wollman MR. Radiotherapy for metastases to the mandible in children. J Oral Maxillofac Surg 2002;60:269–71. 11887137 10.1053/joms.2002.30573

[15] Eley KA Wheeler K Tiam RN. An unusual mandibular mass in a child. Oral Surg Oral Med Oral Pathol Oral Radiol 2013;116:386–91. 22901646 10.1016/j.oooo.2012.03.016

[16] Parker CA Liess BD Gov-Ari E. Metastatic neuroblastoma to the mandible: an unusual presentation. Am J Otolaryngol 2011;32:438–40. 20851501 10.1016/j.amjoto.2010.07.015

[17] Kaste SC Hopkins KP Bowman LC. Dental abnormalities in children treated for neuroblastoma. Med Pediatr Oncol 1998;30:22–7. 9371385 10.1002/(sici)1096-911x(199801)30:1<22::aid-mpo8>3.0.co;2-2

[18] Kramer K Cheung NK. Autologous bone marrow transplantation in children with advanced neuroblastoma. Cancer 1995;76:1295–7. 8630912 10.1002/1097-0142(19951001)76:7<1295::aid-cncr2820760730>3.0.co;2-g

[19] Stokes SH Thomas PR Perez CA. Stage IV-S neuroblastoma. Results with definitive therapy. Cancer 1984;53:2083–6. 6704896 10.1002/1097-0142(19840515)53:10<2083::aid-cncr2820531014>3.0.co;2-s

